# Year in review 2025–2026: A year of progress and purpose

**DOI:** 10.1016/j.stemcr.2026.102954

**Published:** 2026-06-09

**Authors:** Hideyuki Okano

**Affiliations:** 1 ISSCR President 2025-2026

## Main text

**Figure:**
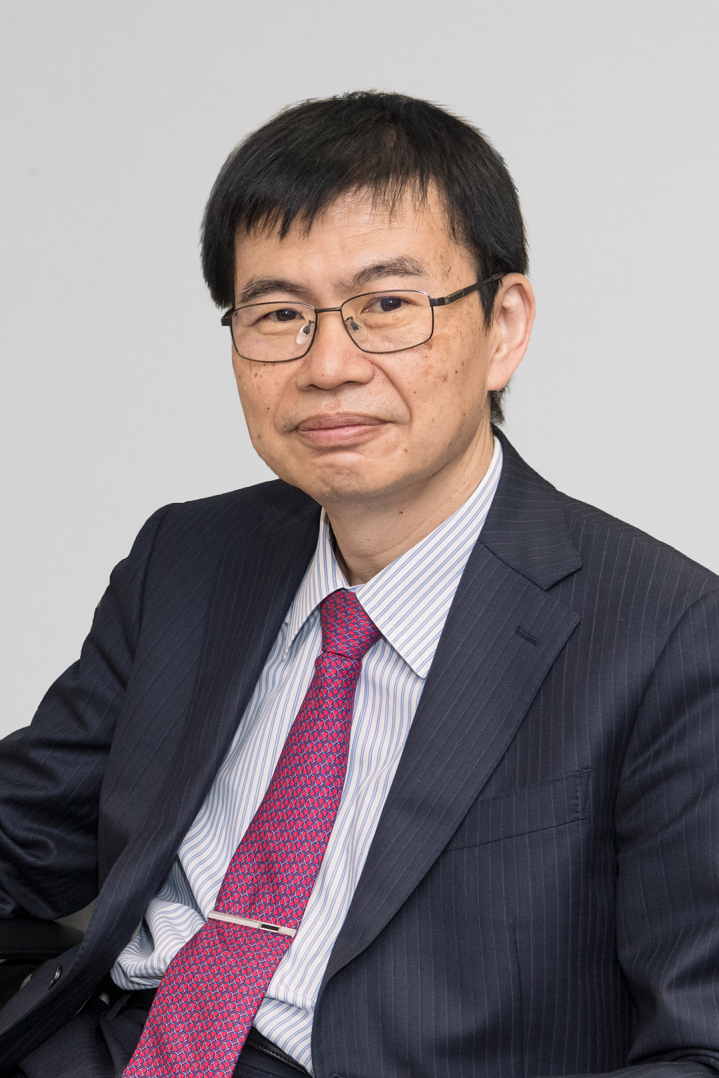
Hideyuki Okano ISSCR President 2025-2026

This past year has been a defining moment not only for the International Society for Stem Cell Research (ISSCR) but also for the field as a whole, and I am proud to have served as ISSCR President at a time of significant progress and reflection. Across the world, stem cell science has continued its transition from potential to practice, with late-stage clinical trials advancing in areas including Parkinson’s disease, age-related macular degeneration (AMD), type 1 diabetes (T1D), severe heart failure, and epilepsy across Australia, Europe, Japan, and the United States.

At the same time, the field has engaged more deeply with complex and often sensitive areas of science, including stem cell-based embryo models, organoids, and the growing need to address access and affordability of emerging cell and gene therapies. These conversations are shaping how science is conducted, translated, and delivered.

Within this broader context, ISSCR plays a central role, not only reflecting the momentum of the field but also actively guiding it. Over the course of my presidential year, the Society has strengthened its scientific leadership, expanded its global reach, and deepened its commitment to responsible innovation. At the start of my term, I defined four themes: expanding scientific programming from the bottom up, bridging basic research and clinical translation, becoming a truly inclusive society, and strengthening communication across leadership and committees. This year reflects meaningful progress across each of these priorities.

### Expanding scientific programming

One of the most visible shifts over the past year is how ISSCR approaches scientific programming. We emphasize building programming that reflects the evolving nature of the field, which is increasingly global, interdisciplinary, and translational.

The ISSCR International Symposia were developed to broaden geographic reach while focusing on areas of accelerating progress, including clinical advances and disease modeling and drug discovery. This ensures that ISSCR programming remained closely aligned with where the science is heading.

A defining moment came with the sold-out symposium, *Accelerating PSC-Derived Cell Therapies: Starting with the End in Mind*, which brought together the global community to highlight the growing momentum behind cell therapy development. Even in the face of industry contraction, the meeting reinforced that the science is advancing and the path toward clinical impact continues to strengthen.

The ISSCR Annual Meeting has also continued to evolve as a platform for new voices. More than 70% of invited speakers have not previously presented at an annual meeting. This reflects an ongoing commitment to elevating emerging leaders and ensuring that the Society’s hallmark event remains dynamic and representative of the field.

This year also saw the launch of new collaborative formats, including an early career-led symposium developed in partnership with the Society for Developmental Biology and the Allen Institute, to take place this fall. By empowering early career scientists to shape scientific dialogue, the ISSCR is building a more participatory and future-oriented programming model.

### Bridging basic research and clinical translation

A defining theme of this year has been strengthening the connection between discovery science and clinical application. The ISSCR is taking meaningful steps to advance pathways that move stem cell research from the laboratory to the patient.

A major milestone was the launch of Best Practices for the Development of Pluripotent Stem Cell-Derived Therapies. This resource reflects years of collective expertise and provides a structured roadmap for navigating translational development, from early research decisions through clinical trials and commercialization.

In parallel, the ISSCR continues to advance its public policy and regulatory efforts to support a robust and responsible environment for stem cell research to flourish. Society leadership and experts have engaged with regulators in India, Korea, Japan, the United Kingdom, and the United States to reinforce the importance of scientifically informed decision-making. The Public Policy Committee hosted its first US Congressional briefing in April, conveying the importance of federal investment in research and vital sources, including human fetal tissue and embryonic stem cells, as the “gold standard” for research in the field. The briefing coincided with an advocacy day and the ISSCR’s role in organizing responses from the US biomedical research community to a National Institutes of Health (NIH) Request for Information (RFI) on reducing reliance on human embryonic stem (hES) cells. The responses, including one from the ISSCR, outline the scientific and clinical need for research with hES cells and encourage NIH to continue to support scientifically meritorious projects in this research area. These efforts are critical to enabling policies that both protect patients and facilitate the development and delivery of advanced stem cell-based therapies and applications.

Complementing public and regulatory policy efforts, the ISSCR Consortium on Advanced Stem Cell-Based Models in Drug Discovery and Development continues to expand, now with two founding members, Bayer and Burroughs Wellcome Fund. This initiative brings together stakeholders from academia, industry, and regulatory sectors to address a critical challenge—advancing fit-for-purpose use of stem cell-derived models in drug discovery and drug development. By focusing on validation frameworks, standards, and data interoperability, the consortium is laying the groundwork for a new generation of preclinical science.

At the core of these advances is recognition of the essential role of basic research. Fundamental discovery science remains the engine that drives the entire field forward, providing insights into cell identity, development, and disease mechanisms that make clinical innovation possible. The ISSCR consistently reinforces this foundation through its scientific programming, standards, advocacy, and publications, because we know that as science accelerates toward the clinic, it is only possible through sustained investment in basic research.

This translational focus also extends to real-world challenges. The ISSCR Summit on Access and Affordability in Cell and Gene Therapies, held in March in collaboration with CIRM and the UCLA Broad Stem Cell Research Center, brought together leaders across sectors to address economic and systemic barriers that may limit patient access. Discussions on pricing, manufacturing, and reimbursement highlighted the complexity of ensuring that scientific advances translate into equitable outcomes.

At the same time, *Stem Cell Reports* is experiencing strong momentum. With one of the highest submission rates in its history in 2025 and the implementation of enhanced rigor and reproducibility standards, including the ISSCR Standards Checklist and STAR Methods, the journal continues to reinforce its role as a leading platform for high-quality science and for discussions on policy and ethics.

### Becoming a truly inclusive society

One of the most forward-looking efforts we launched in November is a global workforce development initiative for stem cell research and regenerative medicine in partnership with Canada’s Stem Cell Network. By bringing together early-career scientists and trainees from around the world, this initiative is helping to shape a more inclusive and sustainable future. It focuses on understanding career pathways, identifying skills gaps, and defining strategies to support the next generation of researchers, something sorely needed at this moment in time with rapid advances in the field and escalating funding uncertainties.

Education also plays a central role in advancing inclusion. Through two continuing education courses in Stem Cell Medicine, developed in collaboration with Harvard Medical School, we have already reached more than 7,000 learners across over 120 countries. We are now building on this momentum with additional disease-specific courses, including one focused on Parkinson’s disease that launched in March. Available in six languages, the CE courses aim to equip clinicians with the knowledge needed to evaluate emerging therapies, engage in informed discussions with patients, and address widespread misinformation about stem cell treatments.

### Strengthening communication across leadership and committees

One of the most impactful changes of my term has been strengthening communication and alignment across the ISSCR’s leadership structure. Deliberate efforts have ensured that the Society’s strategic priorities are consistently reflected across all areas of activity.

A key step has been the integration of board members into each committee, creating a direct connection between leadership and operational initiatives. This has improved the ability to identify emerging issues, align efforts with strategic goals, and cultivate a more cohesive organizational culture.

This year, we increased collaboration among committees as well. Joint efforts, such as the work between the Education and Policy Committees on advocacy messaging, demonstrate how cross-functional engagement can lead to stronger and more unified outcomes. This model will continue to evolve, supporting the Society’s ability to address complex challenges with greater coordination.

Engagement with ethical and policy issues has also deepened through collaborations such as the partnership with the Nuffield Council on Bioethics to conduct a global horizon scan of stem cell research. This work ensures that ISSCR remains proactive in addressing emerging challenges while reinforcing its leadership in responsible scientific advancement.

### A year that positions the future

This past year has been one of significant progress. Equally important, it has sharpened our direction for what comes next.

We know that the pace of discovery in stem cell science continues to accelerate, bringing new scientific, clinical, and policy challenges. These advances require trusted standards, clear guidance, and global coordination to ensure progress is responsible and earns public confidence. At the same time, geopolitical instability, funding pressures, regulatory uncertainty, and the spread of unproven interventions underscore the need for credible, global scientific leadership.

This moment presents both urgency and opportunity. The ISSCR will expand its role as the global authority on standards, consensus guidance, and best practices, while deepening engagement with policymakers and regulators to support scientifically informed decisions and responsible translation. *Stem Cell Reports* will remain central to this effort as the Society’s flagship platform for high-quality research and the exchange of perspectives emerging from ISSCR initiatives and collaborations.

Sustaining progress also requires investing in people. The ISSCR will focus on strengthening the pipeline of future scientists through expanded training, mentorship, and career development, while diversifying the resources that support them.

As global collaboration becomes more complex, the ISSCR will reinforce its role as a convener, connecting scientists, institutions, and partners across borders to advance shared goals.

Together, these priorities underscore a clear imperative to leverage the Society’s authority, global reach, and convening power to guide the responsible advancement of stem cell science and shape its future. The progress of the past year has laid a strong foundation for what comes next—a bold, forward-looking agenda to ensure the field’s full potential is realized for our members, patients, and society.

